# The Effect of Temperature on Moisture Transport in Concrete

**DOI:** 10.3390/ma10080926

**Published:** 2017-08-09

**Authors:** Yao Wang, Yunping Xi

**Affiliations:** Department of Civil, Environmental and Architectural Engineering, University of Colorado Boulder, Boulder, CO 80309, USA; yunping.xi@colorado.edu

**Keywords:** concrete, moisture transport, temperature gradient, coupling parameter

## Abstract

Most concrete structures and buildings are under temperature and moisture variations simultaneously. Thus, the moisture transport in concrete is driven by the moisture gradient as well as the temperature gradient. This paper presents an experimental approach for determining the effect of different temperature gradients on moisture distribution profiles in concrete. The effect of elevated temperatures under isothermal conditions on the moisture transport was also evaluated, and found not to be significant. The non-isothermal tests show that the temperature gradient accelerates the moisture transport in concrete. The part of increased moisture transfer due to the temperature gradient can be quantified by a coupling parameter D*_HT_*, which can be determined by the present test data. The test results indicated that D*_HT_* is not a constant but increases linearly with the temperature variation. A material model was developed for D*_HT_* based on the experimental results obtained in this study.

## 1. Introduction

Moisture transport plays a main role in predicting the durability and serviceability of cement-based materials. For instance, the high moisture content rapidly increases the freeze-thaw deterioration of concrete [1,2,3]. The moisture curing condition strongly affects the hydration degree and strength development for the cement-based materials, like ordinary Portland cement (OPC) concrete, and mortar with various cement-replacements [4,5,6,7,8,9,10,11,12]. In addition, the high level of internal moisture can help to accelerate the chloride penetration in concrete and trigger corrosion of embedded steel bars, especially in marine structures [13,14,15,16,17,18,19].

In order to improve the durability and serviceability of concrete structures, the mechanisms of moisture transfer must be understood well. Many researchers consider that the moisture transfer in concrete is driven by a moisture concentration gradient, and thus the moisture flux is considered as a function of a moisture concentration gradient, and the material parameter associated with the moisture concentration gradient is the coefficient of moisture diffusivity. For the temperature effect, the coefficient of moisture diffusivity was considered as a function of temperature [20,21]. Under the service condition, however, temperature fluctuation and moisture changes in concrete structures occur simultaneously; both temperature and the temperature gradient must be considered in terms of moisture transfer. In the literature, the effect of the temperature gradient on moisture transfer was treated by using a separating term in the moisture flux equation, and this coupling effect was shown to be significant under non-isothermal conditions [22,23].

When the moisture gradient and the temperature gradient exist at the same time, there are two coupling effects. One is the effect of the temperature gradient on moisture transfer, which is called “Soret effect”. The other one is the effect of moisture gradient on thermal conduction, which is not the focus of this study. “Soret effect” is a general term to explain the effect of the temperature gradient on diffusing species, such as salt solutions. It was first discussed by the Swiss scientist Charles Soret (1879) [24]. He discovered that the salt solution in a tube did not remain at a uniform concentration when the two ends of the tube were kept under two different temperatures. The concentration of salt was higher near the cold end than near the hot end. He concluded that a flux of salt was generated by the temperature gradient and the coupling effect was then named as “Soret effect”. In our case, the moisture flux in concrete is not only generated by the moisture concentration gradient but also by the temperature gradient [25,26,27,28,29].

This paper presents an experimental method for evaluating the moisture transfer in concrete under a given temperature gradient, and an analytical approach to determine the coupling parameter for the “Soret effect” of concrete using the obtained test data. The moisture distributions in concrete specimens under four different temperature conditions were studied, including isothermal and non-isothermal conditions. The isothermal conditions were used to study the thermal effect without the temperature gradient and the non-isothermal conditions were for the gradient effect. Finally, an available theoretical model was used together with the obtained coupling parameter to compare the model prediction with the present test data.

## 2. Theoretical Background

In this section, the governing equations for moisture transfer in concrete will be introduced first, including both equations for the isothermal condition and the non-isothermal condition. These equations will be used to determine the coupling parameter for the Soret effect based on the test data obtained in the present study.

### 2.1. Moisture Transport under the Isothermal Condition

Under the isothermal condition, the moisture flux is only related to a single driven force, which is the moisture gradient. According to Fick’s law, the flux of moisture is proportional to the moisture gradient, as shown in Equation (1):

(1) $$J=-D_{H}\frac{\partial H}{\partial x}$$

where, *J* is the flux of moisture (kg∙m^−2^s^−1^), *H* is the pore relative humidity (%), and *D_H_* is the diffusion coefficient dependent on the pore relative humidity (kg∙m^−2^s^−1^). In this study, the moisture condition is represented by the pore relative humidity *H*, which is a combination of liquid water and water vapor in the pore of concrete [26,30]. It has been used by many researchers to simplify the governing equation for moisture transfer in non-saturated concrete. The diffusion coefficient depends on the diffusing species (which is moisture in the present study) and the characteristics of the concrete pore structure. This coefficient varies as a function of time and concrete mix design parameters, such as water-cement ratio and aggregate volume fraction.

Moisture content must be reflected in the conservation of mass:

(2) $$\frac{\partial w}{\partial t}=-\nabla \cdot J+S_{w}$$

where *w* represents the water content, *t* is time, and *S_w_* is the moisture source or sink that is the correction for the water consumed by hydration at early ages or released by dehydration. A larger correction is required to account for the water released by dehydration due to heating. For our case, there is no moisture source or sink in the concrete. Therefore, *S_w_* equals to 0 and the Equation (2) can be simplified:

(3) $$\frac{\partial w}{\partial t}=-\nabla \cdot J$$

After combining Equations (1) and (3), the moisture transport equation under the isothermal condition is expressed:

(4) $$\frac{\partial w}{\partial H}\frac{\partial H}{\partial t}=\nabla \cdot \left(D_{H}\frac{\partial H}{\partial x}\right)$$

where $\frac{\partial w}{\partial H}\frac{\partial H}{\partial t}=\nabla \cdot \left(D_{H}\frac{\partial H}{\partial x}\right)\left(4\right)$ *∂w/∂H* is the moisture capacity, which is the derivative of adsorption isotherm *w*(*H*).

In Equation (4), the moisture capacity can be estimated by available material models, which will be discussed later. Then, the moisture diffusion coefficient is the only unknown which can be determined by concentration profiles of moisture in concrete samples in the present study.

Under the isothermal condition, there is another temperature effect that must be considered—that is, the effect of the temperature level (without the temperature gradient) on moisture transfer. For any mass transfer process, higher temperatures can accelerate the mass transfer process. For concrete, there are three temperature ranges that need to be considered: the ambient temperature range that is about the range of room temperature; the high temperature range, in which all liquid water turns into water vapour; and the low temperature range, in which all liquid water turns into solid ice. In this study, our focus is the first one, the range of room temperature. So, in the following sections, the moisture concentration profiles under different levels of isothermal condition will be tested and the test data will be used to determine the dependence of *D_H_* on the level of temperature in the range of room temperature.

### 2.2. Moisture Transport under the Non-Isothermal Condition

Under the non-isothermal condition, moisture transfer is driven by the moisture gradient, as well as by the temperature gradient. Indeed, if a system at an equilibrium state is subjected to a temperature gradient, a mass motion takes place and the system reaches a new equilibrium state [20,24,31]. The flux of moisture under a non-isothermal condition can be expressed:

(5) $$J=-D_{H}\frac{\partial H}{\partial x}-D_{HT}\frac{\partial T}{\partial x}$$

where *T* is the temperature (*K*) and *D_HT_* is the coupling coefficient for mass diffusion resulting from the thermal gradient (kg∙m^−2^s^−1^*K*^−1^).

After combining Equations (3) and (5), the balance equation under the non-isothermal condition can be described as:

(6) $$\frac{\partial w}{\partial H}\frac{\partial H}{\partial t}=\nabla \cdot \left(D_{H}\frac{\partial H}{\partial x}+D_{HT}\frac{\partial T}{\partial x}\right)$$

In Equation (6), there are three material parameters: the moisture capacity *∂w/∂H*, the moisture diffusion coefficient *D_H_*, and the coupling coefficient *D_HT_*. Among the three material parameters, *∂w/∂H* can be determined by a theoretical model, and *D_H_* can be determined by the test data in the isothermal condition. *D_HT_* is the only unknown that can be determined by the test data obtained under the non-isothermal condition.

Equations (4) and (6) are the two governing equations for the moisture transfer under the isothermal and non-isothermal conditions in concrete. Experimental setups were designed and implemented in the present study so that the test data can be used to determine *D_H_* and *D_HT_*.

## 3. Experimental Procedures

**Figure 1:**
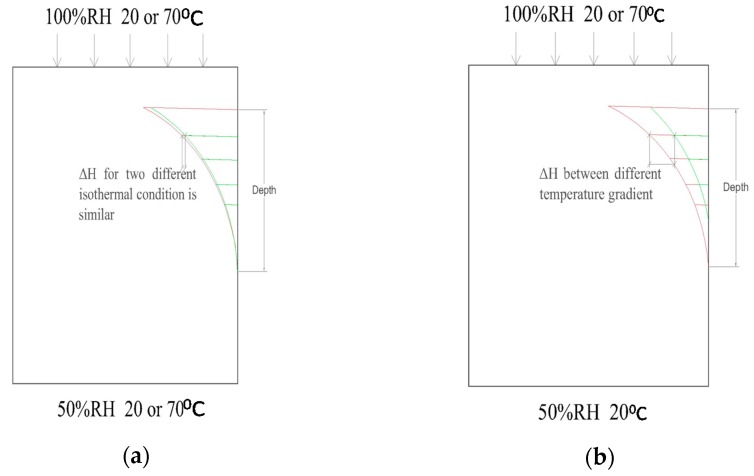
Theoretical model for the study topic: (**a**) isothermal condition test and (**b**) non-isothermal condition test.

The basic ideas of the experimental study are shown in Figure 1. Figure 1a shows the experimental setup of the isothermal test. With different levels of isothermal temperature conditions, the moisture distributions in concrete samples are measured. If they are almost the same, we can conclude that the temperatures in the range of room temperature without a gradient have little effect on moisture transfer.

Figure 1b shows the experimental setup for the non-isothermal test. In this case, both ends of the specimen have the same moisture concentrations but different levels of temperature, so there are temperature gradients in the specimens. If the moisture profile for the higher temperature gradient is higher than the profile from the lower temperature gradient (with the lowest gradient as zero from the isothermal conditions), the effect of the temperature gradient can be identified. In addition, the moisture profiles measured under different temperatures can be used to determine the coupling coefficient *D_HT_*.

### 3.1. Materials and Specimen Preparation

Mix proportions of concrete specimens are shown in Table 1. The cement was Type I Portland cement. The size of coarse aggregate was 0.5 inch (1.27 cm). Fine aggregate was all-purpose sand.

**Figure 2:**
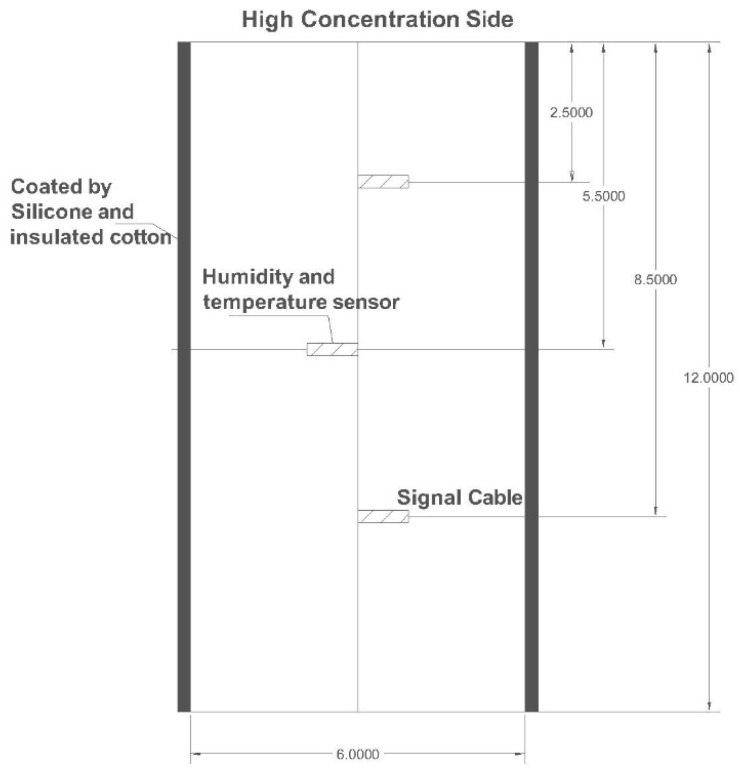
Schematic illustration of the experimental set-up (unit: inch).

The dimensions of the concrete cylinder were 6 inch × 12 inch (15.24 cm × 30.48 cm). They were cured at 23 °C and 100% RH for 28 days and then kept in the room environment for 37 days to reach the same initial conditions with the ambient environment (20 °C and 50%). In order to achieve moisture and temperature transport in one dimension, the samples’ lateral surfaces were sealed by silicone and covered by insulated cotton, leaving the top and bottom sides exposed to ambient air (Figure 2).

### 3.2. Approach to Measure the Internal Relative Humidity and Temperature

**Figure 3:**
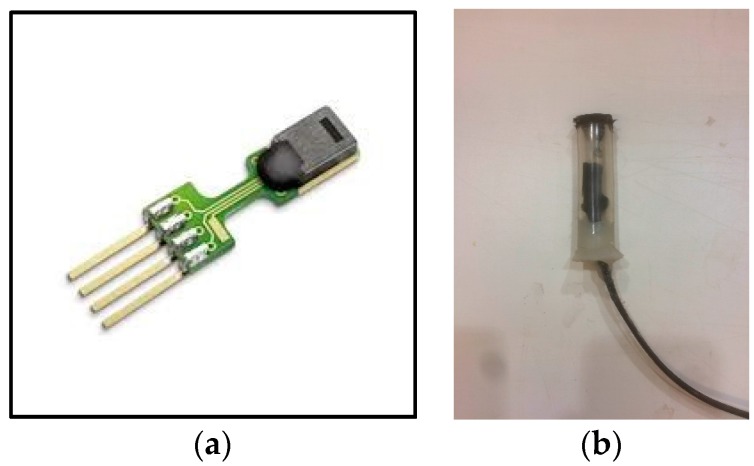
*SHT75* Sensirion humidity and temperature sensor after treatment.

The internal RH and temperature of the specimen was measured by *SHT75* Sensirion humidity and the temperature sensor. The accuracy of the sensor was ±1.8% RH in the range of 10–90% RH at 25 °C. Each sensor was plugged into a plastic tube. Both end sides of the tube were wrapped by GoreTex and the bottom side sealed with silicone (Figure 3). Then, the assemblies were embedded into holes that were drilled into the side of the concrete cylinder. The locations were 2.5, 5.5, and 8.5 inch (6.35, 13.97, 21.59 cm) from the high concentration side (Figure 2).

### 3.3. Experimental Conditions

The environmental condition was around 50% RH and 20 °C. The environmental chamber was used to provide the high isothermal condition, which was set 50% RH and 70 °C. The chamber was controlled by Watlow’s Series F4 1/4 Ramping Temperature Controller (Watlow, St. Louis, MO, USA). The different temperature gradients were generated by the Cole-Parmer EW-03046-20 electric heater (Cole-Parmer, Vernon Hills, IL, USA). The data collection schedule adopted in the study is summarized in Table 2.

## 4. Experimental Results and Discussions

### 4.1. Experimental Results

The samples are named by the experimental condition and boundary temperature. For example, I-70 is the sample under the isothermal condition of 70 °C, while N-60 is the one under the non-isothermal condition (and the top side is heated to 60 °C).

**Figure 4:**
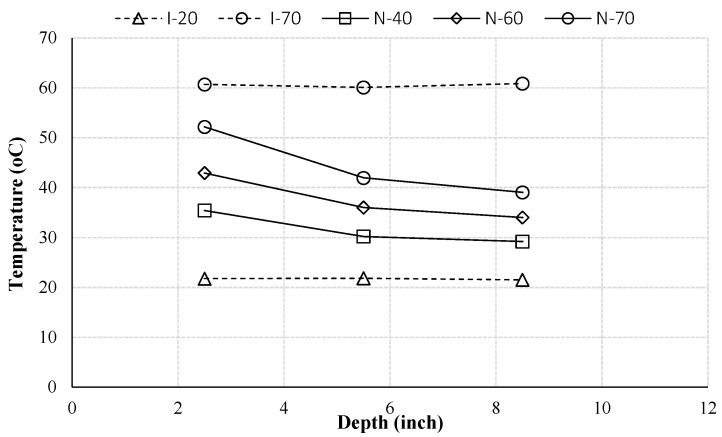
Temperature distribution profiles for all samples at the same time (*t* = 10 day).

Figure 4 shows the temperature distribution profiles in all samples after 10 days. From this figure, one can see that the internal temperatures of I-20 and I-70 both kept uniform at different depths, while the profile of I-70 was higher than I-20. However, the non-isothermal samples were under the temperature gradients. The temperature gradients depend on the boundary conditions. The gradient is higher with a higher temperature on the boundary.

**Figure 5:**
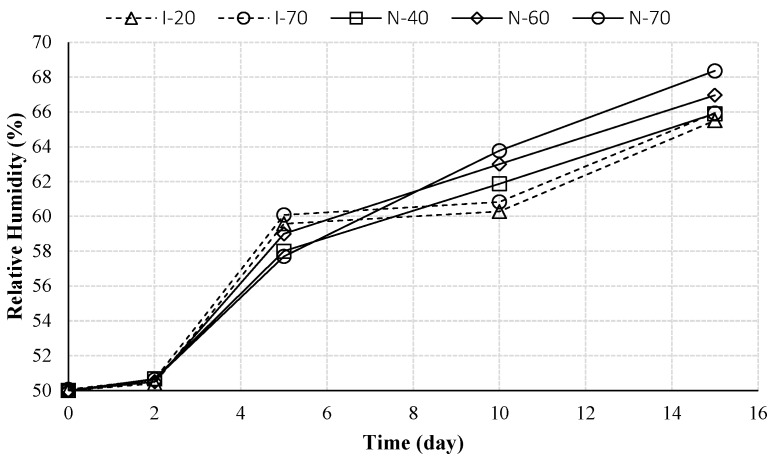
Relative humidity profiles for all samples varies with time increasing at same depth (*x* = 5.5 inch).

Figure 5 compares the moisture profiles in all samples at the same depth of 5.5 inch. As one can see, all samples started from the same initial condition. The moisture profiles of I-20 and I-70 were continuously increased, which resulted from the driving force of moisture gradient [20,21]. Moreover, these two profiles varied in a similar way, indicating that the levels of temperature without a temperature gradient do not have a significant effect on moisture transport. Instead, the moisture profiles of the three non-isothermal cases have different increasing rates and moisture values. The sample with a higher temperature boundary condition has a larger value of moisture level. It indicates that the temperature gradient affects the moisture transfer in concrete under the non-isothermal condition. The same trend was observed in previous studies [22,23].

**Figure 6:**
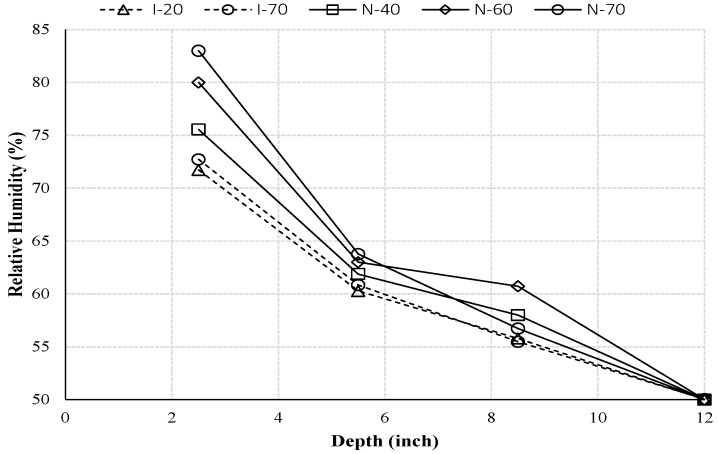
Relative humidity distribution profiles for all samples at the same time (*t* = 10 day).

Figure 6 shows the moisture distribution profiles for all samples after 10 days. From this figure, one can see that at a fixed depth, moisture had a larger value with a higher temperature boundary condition. In addition, a higher temperature gradient resulted in a faster increasing slope of moisture distribution. These results indicate clearly that the temperature gradient is a driving force for moisture transfer [22,23].

Since the effect of the temperature gradient on moisture transfer in concrete is significant, there is a need to consider the contribution of the second term on the right hand side of Equation (6) to moisture transport in concrete, specifically the coupling parameter *D_HT_*.

### 4.2. Evaluation of the Coupling Parameter D_HT_

Equation (6) can be used to evaluate the coupling parameter *D_HT_* based on the experimental results. Using the definition of divergence [32]:

(7) $$\left(J\right)=\frac{A}{V}J=\frac{J}{L}$$

In which Δ*x* is the distance between the sensors. Combining Equations (6) and (7), the balance equation can be rewritten as Equation (8a) and then simplified as Equation (8b):

(8a) $$\frac{\partial w}{\partial t}=\frac{\partial w}{\partial H}\frac{\partial H}{\partial t}=\frac{D_{HH}gradH}{L}+\frac{D_{HT}gradT}{L}=\frac{D_{HH}\Delta H}{L^{2}}+\frac{D_{HT}\Delta T}{L^{2}}$$

(8b) $$\frac{\partial w}{\partial H}\frac{\Delta H\left(t\right)}{\Delta t}=\frac{D_{HH}\Delta H\left(x\right)}{\Delta x^{2}}+\frac{D_{HT}\Delta T\left(x\right)}{\Delta x^{2}}$$

where ∂H = Δ*H*(*x*)/Δ*x* and ∂T = Δ*T*(*x*)/Δ*x* were used. It is apparent that the variation of water content is affected by two driving forces: the moisture gradient and the temperature gradient.

Moisture capacity, ∂*w*/∂*H*, is the derivative of the equilibrium adsorption isotherm. It is related to the water-cement ratio, specimen age, cement type, temperature, and humidity [33,34]. The three-parameter BET model can be used for the equilibrium adsorption isotherm and the corresponding moisture capacity can be obtained as shown in Equation (9), in which the three parameters *C*, *k* and *V_m_* can be characterized by the material models developed by Xi et al., 1994 [33,34].

(9) $$\frac{\partial w}{\partial H}=\frac{CkV_{m}+wk\left[1+\left(C-1\right)kH\right]-wk\left(1-kH\right)\left(C-1\right)}{\left(1-kH\right)\left[1+\left(C-1\right)kH\right]}$$

in which *w* is the moisture content shown in Equation (10).

(10) $$w=\frac{CkV_{m}H}{\left(1-kH\right)\left[1+\left(C-1\right)kH\right]}$$

Parameters *C*, *k* and *V_m_* can be approximately calculated, respectively, as:

(11) $$C=exp\left(\frac{855}{T}\right)$$

(12) $$k=\frac{\left(1-\frac{1}{n}\right)C-1}{\left(C-1\right)}$$

(13) $$V_{m}=\left(0.068-\frac{0.22}{t}\right)\left(0.85+0.45\frac{w}{c}\right)V_{ct}$$

Xi et al., 1994 [33,34,35] also established a model for parameter *n* considering the effects of water-cement ratio, specimen age, cement type, temperature, and humidity:

(14) $$n=N\left(t,w/c,T\right)=N_{t}\left(t\right)N_{wc}\left(w/c\right)N_{ct}\left(c_{t}\right)$$

(15) $$N_{t}\left(t\right)=2.5+\frac{15}{t}$$

(16) $$N_{wc}\left(w/c\right)=0.33+2.2\frac{w}{c}$$

(17) $$N_{ct}\left(c_{t}\right)=\{\begin{array}{c} 1.1TypeI\\ \begin{array}{c} 1.0TypeII\\ 1.15TypeIII\\ 1.5TypeIII \end{array} \end{array}$$

(18) $$N_{T}\left(T\right)=1$$

In Equation (8b), several quantities need to be determined based on the experimental results. Firstly, *D_HH_* can be evaluated depending on the test data from the isothermal case in which there is no effect of the temperature gradient. Secondly, the moisture capacity can be calculated through Equations (9) to (18). Then, Equation (8b) can be rearranged as following for the coupling parameters *D_HT_* [36].

(19) $$D_{HT}=\frac{\Delta x^{2}}{\Delta T\left(x\right)}\left(\frac{\partial w}{\partial H}\frac{\Delta H\left(t\right)}{\Delta t}-\frac{D_{HH}\Delta H\left(x\right)}{\Delta x^{2}}\right)$$

It is important to point out that, in Equation (19), Δ*T*(*x*) and Δ*H*(*x*) are the temperature difference and pore relative humidity difference between two sensors at a fixed time, respectively. Furthermore, Δ*H*(*t*) is the pore relative humidity difference between Δ*t* at a fixed depth. From Equation (19), one can see that the value of *D_HT_* is not a constant.

**Figure 7:**
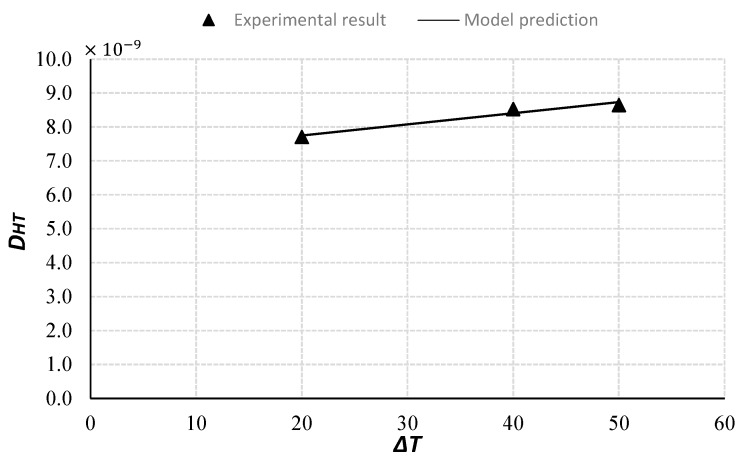
Comparison of experimental results and model predictions of *D_HT_* (%·m^2^·sec^−1^·Celsius^−1^).

Figure 7 presents the values of *D_HT_* for three different temperature gradients. As one can see, the coefficient for the high temperature gradient has a higher value, which means the coupling parameter is temperature dependent. Thus, a simplified model for the coupling parameter *D_HT_* can be derived as a linear function depending on the temperature gradient:

(20) $$D_{HT}=a\Delta T+b$$

The constants *a* and *b* can be determined by a proper curve fitting:

(21) $$D_{HT}=\left(0.0328\times \Delta T+7.0881\right)\times 10^{-9}$$

Figure 7 compares the experimental results and model prediction of the *D_HT_*. They agree reasonably well. It should be mentioned that the proportional constants in Equation (21) depend on concrete mix design parameters, i.e., the water-cement ratio, the aggregate volume fraction, and the ratio of various cement-replacements. A different mix design will result in a different material parameter. Therefore, more systematic studies will need to be conducted to determine the effect of the concrete mix design on the coupling parameter.

### 4.3. Model Verification

The final equation to determine *D_HT_*, Equation (19), was developed based on the governing equation for the coupled moisture heat transfer, Equation (6). In order to verify that Equation (6) can be used to describe the coupled transport processes, Equation (6) ought to be solved with a thermal transfer equation. The coupling parameter *D_HT_* obtained in this study can be used in the solution, and the solution can be compared with the test data. To this end, we assumed that the heat conduction equation is independent from the moisture transfer [37], and we have

(22) $$\rho c\frac{\partial T}{\partial t}=K\frac{\partial^{2}T}{\partial x^{2}}$$

where *c* is the heat capacity, ρ is the density, and *K* is the thermal conductivity. The moisture transfer is governed by Equation (6).

Here we assumed that all material parameters are constants. Some averaged values were taken for the parameters in Equations (6) and (22). The transport processes of heat and moisture are assumed as one dimensional in a very long bar with constant boundary conditions of moisture and temperature, which are consistent with the testing conditions used in this study. By using the Laplace transform and the inverse Laplace transform [38], an analytical solution of the coupled Equation (6) and Equation (22) can be obtained, and the solutions are shown in Equations (23) and (24). The details of the analytical solutions of the two coupled partial differential equations are shown in [39], which will not be described here.

(23) $$T=T_{0}+\left(T_{s}-T_{0}\right)\left(1-erf\left(\frac{x}{2\sqrt{\kappa t}}\right)\right)$$

(24) $$\begin{array}{cc} H_{f}\left(x,t\right)=H_{0}+ & \left(H_{s}-H_{0}\right)\left(1-erf\left(\frac{x}{2\sqrt{d_{HH}t}}\right)\right)\\ & +\frac{d_{HT}\cdot \left(T_{s}-T_{0}\right)}{\left(\kappa -d_{HH}\right)}\left(erf\left(\frac{x}{2\sqrt{d_{HH}t}}\right)-erf\left(\frac{x}{2\sqrt{\kappa t}}\right)\right) \end{array}$$

where,

(25) $$\begin{array}{c} H_{f}\left(x,0\right)=H_{0},\;T\left(x,0\right)=T_{0}\\ H_{f}\left(0,t\right)=H_{s},\;H_{f}\left(\infty ,t\right)=H_{0},\;T\left(0,t\right)=T_{s},\;T\left(\infty ,t\right)=T_{0}\\ d_{HH}=\frac{D_{HH}}{\partial w/\partial H},\;d_{HT}=\frac{D_{HT}}{\partial w/\partial H},\;\kappa =\frac{K}{\rho c} \end{array}$$

subscript 0 is for the initial conditions of temperature and relative humidity, respectively; and subscript *s* is for the surface (boundary) conditions.

**Figure 8:**
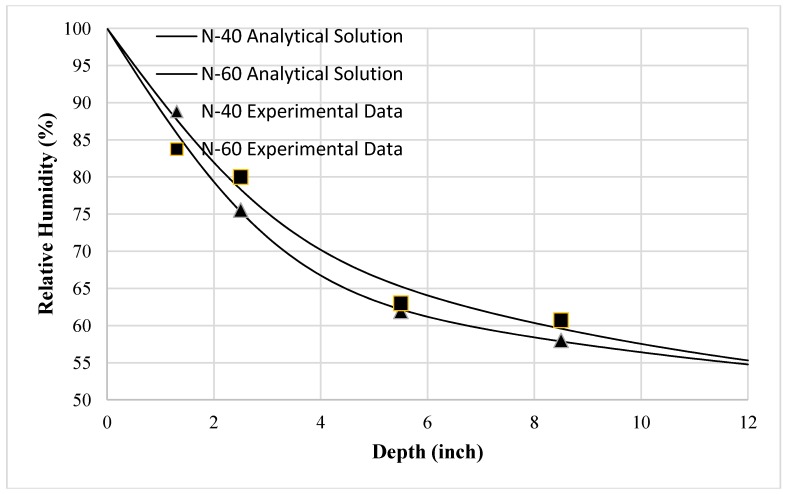
Comparisons of analytical results and experimental data.

Figure 8 shows the comparisons of the analytical results calculated by Equation (24) and the experimental data of the samples. From this figure, one can see that under the given initial and boundary conditions of temperature and moisture, the model predictions of the internal relative humidity distribution profiles agree quite well with the test data. The general trend is that the moisture transfer is increased by the temperature gradient [23,24,26], and that the higher the temperature gradient, the higher the moisture profiles. It indicates that the coupling parameter *D_HT_* obtained in this study can characterize properly the effect of the temperature gradient on moisture transfer.

## 5. Conclusions

(1) A literature review was performed to examine the effect of heat transfer on moisture transfer, the so-called Soret effect. Although the effect was recognized long time ago and studied for different materials, there has been no systematic study for concrete.
(2) An experimental technique was developed for studying the temperature effect on moisture transfer in concrete. The technique was used with four different temperature conditions to study the effect of temperature on moisture transfer in concrete and the test data can be used to obtain the value of the related material parameter.
(3) The experimental data indicates that the elevated temperature without a temperature gradient (an isothermal condition) does not have a significant effect on moisture transfer in concrete. On the other hand, the effect of the temperature gradient under a non-isothermal condition is significant and becomes more significant as the temperature gradient increases.
(4) The effect of the temperature gradient on moisture transfer can be modelled by using an additional term of the temperature gradient in the moisture flux equation. The coefficient of the temperature gradient is called the coupling parameter *D_HT_* (i.e., the coefficient of Soret effect in moisture flux equation).
(5) An analytic approach was developed based on the governing equations for the coupled moisture and heat transfer to calculate the coupling parameter *D_HT_* based on the present test dada. The results show that *D_HT_* is not a constant but increases linearly with temperature variation. In addition, the parameter *D_HT_* depends on the mix design parameters of concrete, such as the water-cement ratio, the aggregate volume fraction, and the ratio of cement-replacement.
(6) An available analytical solution of the coupled moisture and heat transfer equations was used to verify the material parameters obtained in the present study. The predicted moisture distributions under the influence of the temperature gradient were compared with the experimental results. They agreed reasonably well.

## Figures and Tables

**Table 1:** Mix proportions of concrete specimens.

Water–Cement Ratio	Sand-Cement Ratio	Gravel-Cement Ratio
0.6	2.4	2.9

**Table 2:** Experimental strategy adopted.

Specimen	Top Side		Bottom Side	
	RH	*T* (°C)	RH	*T* (°C)
I-20	100%	20	50%	20
I-70	100%	70	50%	70
N-40	100%	40	50%	20
N-60	100%	60	50%	20
N-70	100%	70	50%	20

Note: I stands for isothermal and N stands for non-isothermal.
